# Analysis of retest reliability for pregnant women undergoing cfDNA testing with a no-call result

**DOI:** 10.1007/s11033-023-08591-2

**Published:** 2023-08-03

**Authors:** Shuqiong He, Qian Zhang, Meihuan Chen, Xuemei Chen, Bin Liang, Na Lin, Hailong Huang, Liangpu Xu

**Affiliations:** Medical Genetic Diagnosis and Therapy Center, Fujian Maternity and Child Health Hospital, College of Clinical Medicine for Obstetrics & Gynecology and Pediatrics, Fujian Key Laboratory for Prenatal Diagnosis and Birth Defect, Fujian Medical University, Fuzhou, China

**Keywords:** Cell-free DNA test, Unreportable, No-call, Medical conditions, False-positive rate, Adverse pregnancy outcomes

## Abstract

**Background:**

Determining the reasons for unreportable or no-call cell-free DNA (cfDNA) test results has been an ongoing issue, and a consensus on subsequent management is still lacking. This study aimed to explore potential factors related to no-call cfDNA test results and to discuss whether retest results are reliable.

**Methods and results:**

This was a retrospective study of women with singleton pregnancies undergoing cfDNA testing in 2021. Of the 9871 pregnant patients undergoing cfDNA testing, 111 had a no-call result, and their results were compared to those of 170 control patients. The no-call rate was 1.12% (111/9871), and the primary cause for no-call results was data fluctuation (88.29%, 98/111). Medical conditions were significantly more frequent in the no-call group than in the reportable results group (P < 0.001). After retesting, 107 (107/111, 96.40%) patients had a result, and the false-positive rate (FPR) of retesting was 10.09% (10.09%, 11/109). In addition, placental lesions were more frequent in the no-call group than in the reportable results group (P = 0.037), and 4 patients, all in the no-call group, experienced pregnancy loss.

**Conclusions:**

Pregnant women with medical conditions are more likely to have a no-call result. A retest is suggested for patients with a no-call result, but retests have a high FPR. In addition, pregnant women with a no-call result are at increased risk of adverse pregnancy outcomes. In conclusion, more attention should be given to pregnant women for whom a no-call cfDNA result is obtained.

**Supplementary Information:**

The online version contains supplementary material available at 10.1007/s11033-023-08591-2.

## Introduction

Since the discovery of fetal DNA in maternal plasma and serum, there have been numerous studies examining the application of this finding to the clinical setting [1]. Since 2011, based on next-generation sequencing, the cell-free DNA (cfDNA) test has been an important part of clinical practice. A large number of studies have suggested that the cfDNA test is a potent screening method for fetal aneuploidies, with a higher detection rate (> 99%) and a lower false-negative rate than other currently available screening methods (< 1%) [2–4].

However, unreportable or no-call cfDNA test results still exist. The no-call rate varies among different approaches. According to a previous review, methods based on massive parallel sequencing (MPS) were subtotaled to have the lowest no-call rate (1.58%), followed by chromosome-specific sequencing (3.56%), and the SNP-based method had the highest rate (6.39%) [5]. The meta-analysis of Gil et al. [6] showed three important reasons for such failure: first, problems with blood collection and the transportation of samples to the laboratory, including an inadequate blood volume, hemolysis, the incorrect labeling of tubes and delays in arrival to the laboratory; second, low fetal fractions (FFs), usually below 4%; and third, assay failure due to a variety of reasons, including failed DNA extraction, amplification or sequencing.

Several studies have explored the factors related to no-call test results. A prospective study of 23,495 pregnant women who underwent cfDNA testing showed that maternal age, weight, racial origin and parity, gestational age, method of conception, serum pregnancy-associated plasma protein-A levels, and free β-human chorionic gonadotropin levels are independent predictors of no-call cfDNA test results [7]. In the retrospective analysis of Liu et al., the women with a no-call cfDNA test result were divided into a high-risk group and a low-risk group according to maternal age and maternal serum screening (MSS). They demonstrated that high-risk pregnant women with a no-call cfDNA test result are at increased risk of adverse pregnancy outcomes [8].

Although the above two studies reported the correlative factors of no-call test results, they did not take into account the underlying medical conditions of the mothers. Another retrospective analysis by Dabi et al. showed that the presence of an autoimmune disorder, but not repeated vein thrombosis or other chronic conditions, in pregnant women was associated with nonreportable cfDNA test results [9]. Research from MacKinnon et al. also verified that women with autoimmune diseases have higher rates of indeterminate cfDNA test results than women without autoimmune diseases [10]. However, this research did not discuss any subsequent management. Notably, a no-call test result makes genetic counseling difficult. A consensus on the subsequent management of patients with a no-call cfDNA test result has not yet been reached. Another issue that needs to be discussed is whether a retest is necessary and whether the outcomes from a retest are reliable.

In this study, we retrospectively analyzed 9871 women with singleton pregnancies undergoing cfDNA testing at a single tertiary center in Fujian Province, southeastern China, in 2021. Among these pregnant women, 111 obtained a no-call test result after the first sequencing. This study aimed to explore the potential factors related to no-call cfDNA test results and to discuss retest reliability. Clarifying these problems could be favorable for choosing follow-up management of these pregnant women.

## Materials and methods

### Subjects

The study included 9871 women with singleton pregnancies who underwent cfDNA testing at a single tertiary center in Fujian Provincial Maternity and Children’s Hospital in 2021. Among these pregnant women, 111 obtained a no-call result after the first sequencing at the first sample. And then we used two systems to select the control group. One system contained the cfDNA test results, and the other contained the maternity results. First, in the previous system, data from 9760 patients with a reportable cfDNA test result were exported to Excel and numbered according to the time of blood collection. Second, based on the 1:2 ratio of the no-call and reportable results groups, 222 patients with reportable results were randomly selected from the 9760 patients. However, of the selected 222 patients with reportable results, 42 did not deliver in this hospital, 9 lacked the required clinical data, and 1 was excluded for having a vanishing twin. Therefore, the reportable results group ultimately included 170 patients. To sum up, 111 patients received a no-call result and were compared with 170 control patients.

### cfDNA test

The integral process of a cfDNA test includes cfDNA preparation and sequencing. Every step involves strict quality control. The process of cfDNA preparation mainly includes blood collection, sample transportation, plasma separation, cfDNA extraction, cfDNA library construction and library quantification. Samples were collected and tested in the same hospital. For each patient undergoing cfDNA testing, 10 mL of peripheral venous blood was collected in an EDTA anticoagulant tube and separated through a double centrifugation procedure within 96 h, and those with problems of inadequate blood volume, hemolysis, incorrect tube labeling and delays in arrival to the laboratory were excluded; otherwise, a redraw assay was needed. For those collected before 4:00 p.m., plasma separation was completed on the same day. Samples collected after 4:00 p.m. were kept at 4 °C overnight, and plasma separation was completed by 12:00 a.m. the following day. The plasma was frozen at ≤ −20 ℃ if not processed immediately. According to the manufacturer’s instructions, cfDNA was extracted via a DNA extraction and purification kit (Berry Genomics Corporation), and the concentration was measured via a Qubit 2.0 fluorimeter (Thermo Fisher Scientific). A repeat assay was performed for samples with a concentration of cfDNA > 0.7 ng/µL. Follow-up DNA library preparation, purification, sequencing, and data analysis were performed with the Bambni™ assay (Berry Genomics Corporation). The library concentration was quantitated by a fluorescence quantitative polymerase chain reaction instrument (Applied Biosystems StepOnePlus), and the quality control included the following: -3.6 ≤ slope ≤ -3.1, R^2^ ≥ 0.99, 90% ≤ reaction efficiency ≤ 110%, no template control with nonspecific amplification, and a concentration of the cfDNA library ≥ 10 pM; otherwise, a repeat assay was needed. Only when these pre-analytical errors and laboratory errors were under control was sequencing be carried out.

The NextSeq CN500 sequencing platform (Berry Genomics Corporation) was used for MPS. Sequencing quality control included data filtering, original quality control, analytical quality control and GC correction, statistical analysis and manual review. In original quality control, the number of bases with a sequencing error rate ≤ 1‰ should be greater than 80%. Next, the sequencing reads were aligned to the human genome sequence (hg19) and processed with a Z score-based Bambi data analysis system. Only the sequencing reads aligned to just one location in the human genome sequence with no mismatch could be counted, and this was named a ‘unique map read’, their guanine-cytosine (GC) content was calculated. In analytical quality control, the unique map reads must be greater than 1.5 Mb. For any given chromosome in each sample, the normalized chromosome representation (NCR) value was calculated as follows: the count of the sequences uniquely mapped to the chromosome of interest/the total count of the sequences uniquely mapped to all the autosomal chromosomes [11]. For all chromosomes except chromosome 14 and chromosome Y, the NCR values were plotted against the GC content, and the slope was calculated by simple linear regression. The NCR was further normalized by GC content as follows: NCR− (GC−GC_average_ref_)/ Slope_ref_, where GC was the chromosome GC content of the test sample, and GC_average_ref_ and Slope_ref_ were the average values of the reference samples, respectively. Finally, the Z score of each chromosome was calculated. For all chromosomes except chromosome 14 and chromosome Y, the Z score for chromosome i in the test case was calculated as follows: ((NCR_gc_i in the test case) − (mean NCR_gc_i in the reference controls))/ (standard deviation of NCR_gc_i in the reference controls). For chromosome 14 and chromosome Y, the Z scores were calculated without GC correction. Chromosomes with a Z score between −3 and 3 were considered to have a low risk of aneuploidy, whereas chromosomes with a Z score ≥ 3 were considered to have a high risk of trisomy, and chromosomes with a Z score ≤ −3 were considered to have a high risk of monosomy or microdeletion [12]. Results for all samples were reported within 10 working days of collection. Samples were stored at −80 °C as soon as possible after reporting.

### Criteria for no-call cfDNA test results

In this study, a no-call cfDNA test result was defined as a nonreportable result for the first sample after the first sequencing. Accordingly, the reportable results group, i.e., the success group, had a reportable result for the first sample after the first sequencing. A repeat or redraw assay was performed for the original or redraw sample according to the suggestion of the analysis system when faced with a no-call test result. The situations of no-call test results were as follows: first, a low FFs, which was defined as < 3%; second, data fluctuations, pointing to Z score fluctuations, which were caused by sample degradation, library preparation, or other technical reasons. Z score fluctuation suggested that the sample exhibited significant multiple chromosomal aneuploidies, most likely due to test failure and not chromosomal aneuploidies.

### Karyotyping analysis

The process of karyotyping analysis, including cell culture and G-banded karyotyping, was performed according to standard procedures. Karyotypes were described according to the International System for Human Cytogenetic Nomenclature.

### Chromosomal microarray analysis (CMA)

The genomic DNA (gDNA) of the fetus was extracted from amniotic fluid by using a QIAGEN kit according to the manufacturer’s instructions. Next, the gDNA was digested, ligated, amplified, purified, fragmented, labeled, hybridized, stained, and scanned according to the standard operating procedure of the Affymetrix CytoScan 750 K GeneChip (Affymetrix). Chromosome Analysis Suite V3.2 software was used for data analysis. Publicly available databases, including the Database of Genomic Variants, the DECIPHER Database, Online Mendelian Inheritance in Man, the International Standards for Cytogenomic Arrays, PubMed, and ClinGen, were searched for the detected copy number variations (CNVs). The human genome version GRCh37 (hg19) was used for annotation. CNVs larger than 400 kb and a loss of heterozygosity (LOH) ≥ 10 Mb were considered.

### Statistical analysis

IBM’s Statistical Package for the Social Sciences (SPSS) statistics for Windows (version 20.0) (IBM corporation, Armonk, NY, USA) was used for the statistical analysis of the collected data. A normality test (Shapiro-Wilk test) was performed for 8 continuous variables, the results suggested that of all the continuous variables, only the data for age and height conformed to a normal distribution (supplement Fig. S1). The Student’s t test was used for these two parametric continuous data (supplement Fig. S2). For the nonparametric continuous data, the Mann-Whitney U test was performed (supplement Fig. S3). The Chi-squared test was used for categorical variables. P < 0.05 was considered statistically significant.

## Results

### Study population

Of the 9871 women with singleton pregnancies undergoing cfDNA testing during the inclusion period, 111 had a no-call result after the first sequencing, and 170 were included as the control group. The maternal characteristics of the 281 pregnant women are summarized in Table 1.

There was no significant difference in the average maternal age, weight, height, BMI, or mode of conception between these two populations. The gestational age of the no-call group was slightly higher than that of the reportable results group ((P = 0.040). In addition, underlying medical conditions were distinctly more frequent in the no-call group than in the reportable results group (P < 0.001), whereas there were no significant differences for specific diseases.

**Table 1:** Maternal characteristics of the 281 women with singleton pregnancies

Characteristics	Reportable results group (n = 170)	No-call group (n = 111)	P value
Maternal age	31.73 (29–35)	31.90 (28–36)	0.759
Maternal weight (kg)	68.32 (62-74.58)	68.32 (62.9-73.55)	0.732
Maternal height (m)	1.61 (1.57–1.65)	1.60 (1.57–1.64)	0.900
Maternal BMI	26.50 (24.26–28.74)	26.57 (24.59–28.63)	0.915
Gestational age at the first sampling	16.79 (14.71-18)	17.35 (15-18.29)	0.040^*^
Mode of conception			0.242
Spontaneous	162 (95.29%)	102 (91.89%)	
Assisted	8 (4.71%)	9 (8.11%)	
Medical condition	63 (37.06%)	47 (42.34%)	< 0.001^*^
GDM	33 (19.41%)	27 (24.32%)	0.326
Autoimmune disorders	12 (11.86%)	7 (6.31%)	0.806
Endocrinopathy	10 (2.82%)	8 (7.21%)	0.657
Thrombophilia	6 (3.95%)	5 (4.50%)	0.680
Others^a^	17 (10.00%)	13 (11.71%)	0.650

Data are presented as the mean (interquartile range) or n (%)

BMI: body mass index; GDM: gestational diabetes

^a^ Including chronic hypertension, gestational hypertension, pulmonary arterial hypertension, preeclampsia, intrahepatic cholestasis of pregnancy (ICP), obesity, hypoproteinemia, cytomegalovirus infection, appendicitis, chronic viral hepatitis B, and recurrent miscarriage (RM)

^*^ P < 0.05

### Subsequent management of patients with no-call test results

When a no-call result was received, the analysis system suggested subsequent processing, including a repeat assay (from the original sample) or a redraw assay (from the redraw sample), depending on the causes of the no-call result (Table 2). A repeat assay was performed for 99 patients (89.19%, 99/111), and data fluctuation was the primary cause (98.99%, 98/99). A redraw assay was performed for another 12 (10.81%, 12/111) patients, mainly because of low FFs (41.67%, 5/12) and sex chromosome anomalies (SCAs) (41.67%, 5/12).

**Table 2:** Causes of no-call test results and suggestions for subsequent processing

Indication for subsequent processing	Causes of no-call test result	No-call (n = 111)
Repeat assay n = 99 (89.19%, 99/111)	Data fluctuation Less Data/Low map	98 (99.99%, 98/99) 1 (1.01%, 1/99)
Redraw assay n = 12 (10.81%, 12/111)	Low FFs SCAs Multi	5 (41.67%, 5/12) 5 (41.67%, 5/12) 2 (16.67%, 2/12)

Multi: data fluctuation for more than two chromosomes

For repeat assays, a second cfDNA test of the original sample was conducted, and results were obtained in all cases. For redraw assays, the patients were called for a redraw, and then the second cfDNA test was performed using the new sample. A result was received in 8 cases, and the remaining 4 cases failed again. In summary, through retesting, results were obtained for 107 (107/111, 96.40%) patients, including 94 (94/107, 87.85%) patients with normal results and 13 (13/107, 12.15%) with abnormal cfDNA test results, while results could not be obtained for the remaining 4 (4/111, 3.60%) patients.

**Fig. 1:**
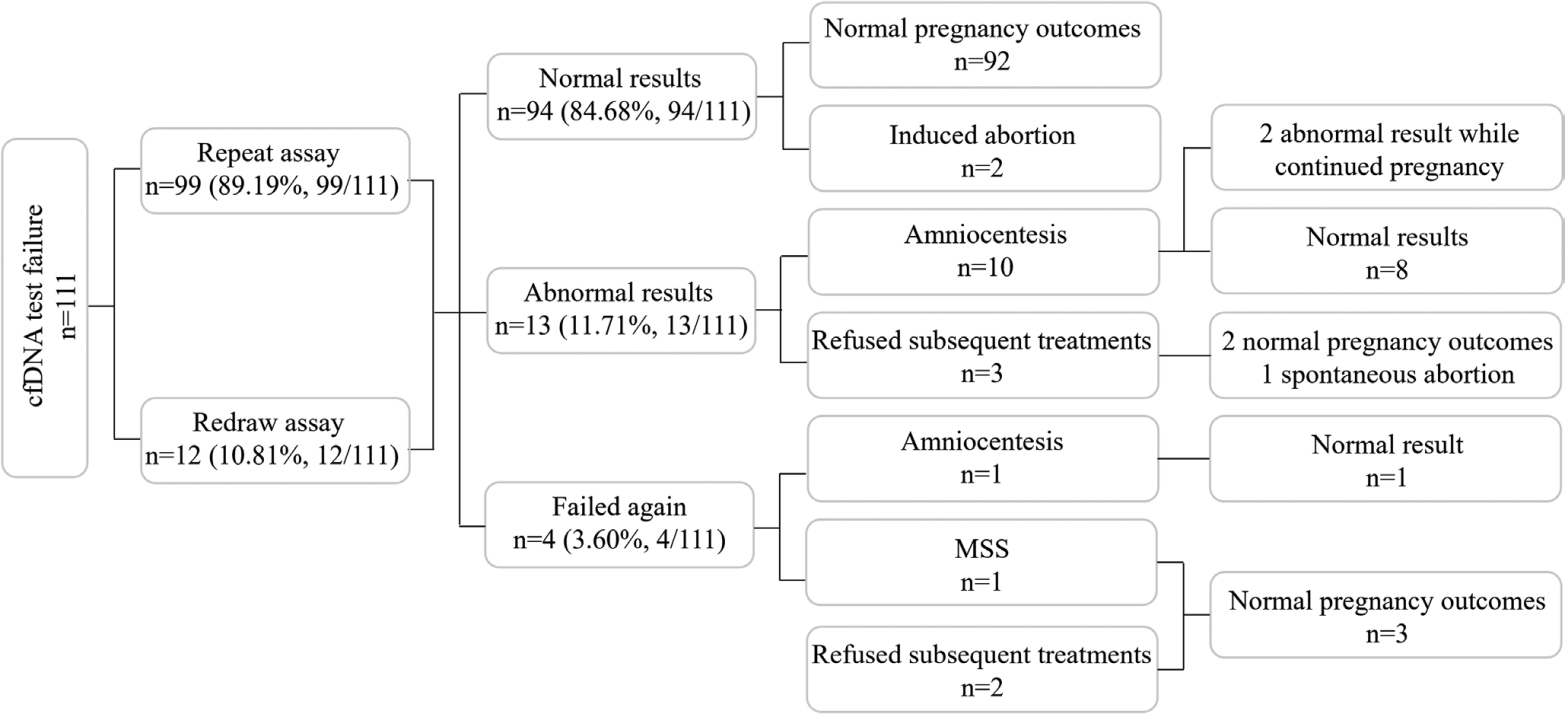
Flowchart of patients with no-call test results and their pregnancy outcomes

These 4 patients underwent posttest counseling; 1 underwent invasive prenatal diagnosis (IPD) testing and received a normal result, 1 chose to undergo second-trimester MSS, and 2 refused any subsequent treatments. All of these patients had normal pregnancy outcomes (Fig. 1).

Of the 94 patients with normal cfDNA test results, none underwent subsequent IPD testing. Among these patients, 2 chose induced abortion due to ultrasound abnormalities, and the remaining 92 had normal pregnancy outcomes.

Among the 13 patients with abnormal cfDNA test results, 10 (10/13, 76.92%) underwent IPD after genetic counseling. Abnormal results were detected in 2 patients, but all had unaffected pregnancy outcomes. Eight patients had normal results, but 1 of them chose to undergo induced abortion due to a fetal structural abnormality detected by ultrasound. The false-positive rate (FPR) of retests was 10.09% (10.09%, 11/109). The remaining 3 patients refused any subsequent treatments; 2 had unaffected pregnancy outcomes, and 1 had a spontaneous abortion. These results are presented in Table 3.

**Table 3:** Subsequent prenatal genetic diagnostic test results for patients with abnormal cfDNA test results

No.	cfDNA test results	Ultrasound findings	Fetal karyotyping	CMA results	Pregnancy outcome
20FJ00486	Trisomy 21	Undetected	/	/	Live birth
20FJ09530	Trisomy 21	Undetected	/	/	Live birth
21FJ07337	Trisomy 21	Undetected	Undetected	Undetected	Live birth
21FJ01968	Monosomy 18	Undetected	Undetected	Undetected	Live birth
20FJ10930	Monosomy 18	Low FL/BPD and FL/HC ratio	Undetected	arr[hg19] 18p11.32p11.31 (2,186,353-5,675,587) × 1	Live birth
21FJ01097	Trisomy 18	Undetected	Undetected	Undetected	Live birth
20FJ10341	Trisomy 18	Mild tricuspid regurgitation	Undetected	Undetected	Live birth
21FJ08259	Trisomy 18 and monosomy X	Undetected	Undetected	Undetected	Live birth
21FJ07999	Trisomy 13	Undetected	/	/	Spontaneous abortion
21FJ04933	Monosomy 13	Short FL and HL, echogenic bowel	Undetected	Undetected	Induced abortion
20FJ10855	Monosomy X	Undetected	Undetected	Undetected	Live birth
20FJ10113	Monosomy X	Undetected	46,X,Idic(Y)(q11.22)	arr[hg19] Yp11.32p11.31 q11.221 (118,551 − 19,556,683) × 2, Yq11.222q11.23 (20,116,737 − 28,799,654) × 0, Yq12 (154,941,868 − 155,233,098 or 59,044,874 − 59,336,104) × 1	Live birth
21FJ00787	Monosomy 1 and 15	Undetected	Undetected	Undetected	Live birth

BPD: biparietal diameter; FL: femur length; HC: head circumference; HL: humerus length

### Pregnancy outcomes

The main pregnancy outcomes are presented in Table 4. The infant lengths were significantly shorter in patients with no-call test results than those of their counterparts with reportable cfDNA tests (P = 0.020). In addition, placental lesions were more common in the no-call group than in the reportable results group (P = 0.037). Moreover, the no-call group had a higher incidence of pregnancy loss than the reportable results group (P = 0.013).

**Table 4:** Pregnancy outcomes

	Reportable results group (n=170)	No-call group (n = 111)	P value
Weeks of gestation at birth	38.93 (38.29-40)	38.78 (38.43-40)	0.545
Infant weight (g)	3209.38 (2996.25–3470)	3141.49 (2950–3390)	0.305
Infant length (cm)	49.63 (50–50)	48.91 (48–50)	0.020^*^
Placental lesions	2 (1.18%)	6 (5.41%)	0.037^*^
Abnormal pregnancy outcomes	2 (1.18%)	6 (5.41%)	0.037^*^
Chromosomal abnormalities	2 (1.18%)	2 (1.80%)	0.655
Pregnancy loss	0	4 (3.60%)	0.013^*^
Spontaneous abortion		3 (2.70%)	
Induced abortion		1 (0.90%)	

Data are presented as the mean (interquartile range) or n (%)

^*^ P < 0.05

## Discussion

Here, we report a retrospective study focusing on pregnant women undergoing cfDNA testing with a no-call result. The no-call rates of the cfDNA tests were assessed by various technologies, and methods based on MPS were subtotaled to have the lowest no-call rate (1.58%) [5]. The no-call rate in our center based on the MPS platform was 1.12% (111/9871), which is lower than that in previous studies [5, 8, 13, 14]. The most important reason is that the no-call rate in the current study was calculated based on whether a result was obtained after sequencing, and problems of previous processes, including sampling, delivery, tracking, and plasma preparation, were not considered. Another reason is probably that the blood samples were collected at later gestational weeks than in the previous studies and therefore had high FFs. In addition, a systematic review suggested lower no-call rates in Asian (0.6%) than in Western (3.3%) populations, which might be due to the lower proportion of obesity in Asian women than in Western women [15]. Obesity is associated with low FFs. In the current study, only 3 (2.70%, 3/111) women with obesity were included in the no-call group, which was lower than the corresponding number in Western countries (55.7%) [16].

Many factors, mainly technical and biological factors, might be associated with no-call test results. In this study, a no-call result was defined as a nonreportable result due to data fluctuation, low FFs, or SCAs, among other factors. The primary cause of no-call test results in the current study was data fluctuation, mainly due to sample degradation, library preparation, and other technical reasons. However, previous studies have demonstrated that the most common reason for no-call test results is low FFs [7, 8]. Different definitions of test failure or no-call test results should be considered. In this study, a no-call test result was defined as a nonreportable result after the first sequencing. A previous study defined test failure as failure in the repeat assay of the original sample [8]. In another study, samples with insufficient fetal DNA were classified as insufficient, and those failing all other laboratory quality metrics, including library and sequencing passing criteria, were classified as having other not reportable etiologies; the results concluded that insufficient quantity represented 0.9% of the samples and that other nonreportable results represented 1.0% of all samples, which suggested that the most common cause of test failure in that study was not low FFs [17]. Therefore, different definitions of test failure or no-call test results might underlie the discrepancy in the primary cause of test failure.

The gestational age of the no-call group was slightly higher than that of the reportable results group, and the primary cause of no-call test results in the current study was data fluctuation. Therefore, the results indicated that a slightly higher gestational age did not reduce the occurrence of data fluctuation. A previous study also showed that gestational age has a negligible influence on no-call test result rates [18]. However, due to the influence of gestational age on the fetal free DNA concentration [19], it is still necessary to collect blood at the appropriate gestational week.

In a pregnant woman’s plasma, the majority of cfDNA is derived from the mother; therefore, the physical condition of the pregnancy will affect the result considerably. Various studies have elucidated that autoimmune diseases [9, 20], ICP [21], heparin administration [22], pre-eclampsia [23], and vanishing twins [24, 25] are associated with nonreportable cfDNA test results. In our cohort, there were significantly more underlying medical conditions in the no-call group than in the reportable results group (P < 0.001), but there was no significant difference for specific diseases. We will next enlarge the sample to explore the impact of specific diseases on no-call test results.

Consensus has not been reached among professional societies on clinical management when faced with a no-call test result. According to the statement of the American College of Medical Genetics and Genomics, a repeat blood draw is not recommended for a no-call test result due to a low FFs if maternal blood is drawn at an appropriate gestational week for noninvasive prenatal screening [26]. However, this claim is not suitable for this study because the primary cause of no-call test results was data fluctuation. In our cohort, a repeat assay or a redraw assay was first conducted according to the indication of the analysis system. In the no-call group, a repeat assay was performed for 99 patients (89.19%, 99/111), and a redraw assay was performed for the remaining 12 (10.81%, 12/111) patients. For the repeat assay, results were obtained in all cases, and the success rate was 100% (100%, 99/99). For the redraw assay, 8 patients received a result, and the success rate was 66.67% (66.67%, 8/12), which is in line with that of previous studies [27–30]. According to our results, a repeat assay has a higher success rate than a redraw assay. In general, 107 patients had a cfDNA test result, with a total success rate of 96.40% (96.40%, 107/111). These results suggested that a repeat assay is necessary if the gestational week allows it.

Of the remaining 4 patients with repeat test failures, 1 underwent prenatal testing and had normal results, 1 chose to undergo second-trimester MSS and had a low-risk result, and the remaining 2 refused any subsequent treatments after posttest counseling. The pregnancy outcomes of these 4 women were unaffected. Chang et al. suggested that low-risk patients (consisting of women with an intermediate-risk MSS result and those who had no risk factors, namely, those aged younger than 35 years who directly requested the cfDNA test) could choose to undergo MSS as an alternative aneuploidy screening method when a no-call test result is obtained at the first sampling [8].

Of the 107 patients with a result, including 94 (94/107, 87.85%) with normal results, none of them underwent subsequent prenatal testing. Abnormal pregnancy outcomes were seen in two patients, including one fetus with cerebellar hypoplasia and another with intrauterine infection. The pregnancy outcomes of the remaining 92 patients were unaffected.

Of the remaining 13 (13/107, 12.15%) patients with abnormal cfDNA test results, repeat assays were performed for all of them, including 6 with trisomy 21/18/13, 3 with monosomy 18/13, 2 with SCAs, 1 with monosomy 1 and 15, and 1 with trisomy 18 and monosomy X. After being offered genetic counseling, 10 (10/13, 76.92%) patients underwent prenatal testing of amniotic fluid or cord blood, and 3 refused any subsequent management. The uptake of invasive testing after receiving an abnormal result in this study was consistent with that in our previous result (76.92% vs. 77%) [31]. Of the 10 patients who underwent prenatal testing, 2 had abnormal prenatal genetic results, and the remaining 8 had cfDNA false-positive results. In one patient with abnormal prenatal genetic results, a CMA test showed a 3.4 Mb deletion in the 18p11.32p11.31 region, and ultrasound revealed low FL/BPD and FL/HC ratios in the fetus. In another case, the fetal karyotype analysis result was 46,X,Idic(Y)(q11.22), and the CMA test suggested a 19.4 Mb gain at the region of Yp11.32p11.31q11.221, an 8.7 Mb deletion at Yq11.222q11.23 and a 291 kb deletion at Yq12. These 2 families were informed of the results and chose to continue the pregnancy, with a live birth outcome.

The FPR for retests was 10.09% (10.09%, 11/109), and in the reportable results group, it was 0% (0%, 0/168). This result suggested that the FPR of the no-call group was higher than that of the reportable results group, which is in line with a previous study [5, 32]. However, the FPR of the retests in this study was higher than that reported (10.09% vs. 3.65%). This nonconformity may also be due to the different definitions of cfDNA test failure or no-call test results. According to Zhang et al., a no-call test result was considered when the concentration of cfDNA was > 0.7 ng/µL [32]. They pointed out that if a no-call test result was caused by a high cfDNA concentration, the second test may fail to obtain a result, thus avoiding reaching a false-positive result to a certain extent. This criterion was not used in this study. According to our results, retesting is necessary to alleviate anxiety, and IPD should be offered when a positive result is obtained.

Among the remaining 8 patients who underwent prenatal testing with normal results, 1 chose to undergo induced abortion due to structural abnormalities, including a short FL and echogenic bowel shown in the fetus via ultrasound. This patient had underlying medical conditions, including gestational diabetes, autoimmune disorders, chronic hypertension, and RM. Whole-exome sequencing (WES) or genome sequencing (GS) was suggested for this patient before the next pregnancy. The diagnostic yield of WES in anomalous fetuses with normal karyotype and CMA results was 39.4% [33]. In addition, a study by Dong et al. demonstrated that low-pass GS enables the identification of RM-related chromosomal abnormalities at a higher diagnostic yield and resolution independent of routine chromosome analysis [34]. With the utilities of WES and GS, patients can gain information on the guidance and assessment of recurrence risk for the subsequent pregnancy. The pregnancy outcomes of another 7 patients were unaffected. The remaining 3 patients refused any subsequent management, including 2 with live births and 1 with spontaneous abortion due to intrauterine infection.

According to our results, the infant size was significantly shorter in the no-call group than in the reportable group. This may be due to the 4 patients with pregnancy loss at lower gestational weeks, thus resulting in a shorter infant length in the no-call group because there was a nonsignificant difference between these two populations when the above 4 patients were excluded.

Although the sample size was small, the results showed that no-call test results were associated with a higher rate of placental lesions (5.41% vs. 1.18%). Juul et al. demonstrated that cfDNA fragments are released from placental trophoblasts into the maternal bloodstream [15]. An earlier study indicated that circulatory fetal nucleic acids may be viewed as new tools to study alterations in placentation [35]. Further evidence is needed to explore whether placental lesions affect the results of cfDNA tests.

Several studies have demonstrated that no-call test results are related to adverse pregnancy outcomes [8, 9, 32]. In our cohort, the no-call group had a higher incidence of adverse pregnancy outcomes than the reportable results group (5.41% vs. 1.18%), which is inconsistent with a previous study. Additionally, there was a significant difference in the pregnancy loss rate between the reportable results and no-call groups (3.60% vs. 0%). The rate of pregnancy loss was similar to that in another retrospective analysis in which the fetal loss rate was 5.47% (15 out of 274 patients with no-call results experienced fetal loss) [13]. Therefore, attention should be given to patients with a no-call cfDNA test result. Careful diagnosis and appropriate medical intervention are necessary to avoid adverse pregnancy outcomes as much as possible.

## Conclusions

Our study revealed that pregnancies with underlying medical conditions tend to have a no-call result after undergoing cfDNA testing. Further study with larger samples could help to elucidate the specific diseases related to no-call test results. A retest can significantly reduce the no-call rate but has a high FPR. Therefore, retesting is suggested when the gestational age allows it, and positive findings should be verified by invasive prenatal diagnosis. In addition, a no-call result is associated with a higher risk of adverse pregnancy outcomes, especially pregnancy loss. Therefore, these patients need careful counseling and timely and appropriate medical intervention to prevent poor outcomes.

## Electronic supplementary material

Below is the link to the electronic supplementary material.

Supplementary Material 1

## Data Availability

The datasets generated during and/or analyzed during the current study are not publicly available, but are available from the corresponding author on reasonable request.
