# Supplementary material for: Analysis of retest reliability for pregnant women undergoing cfDNA testing with a no-call result
Source: Mol Biol Rep. 2023 Aug 3;50(9):7649–57. doi: 10.1007/s11033-023-08591-2 (PMC10460704; doi:10.1007/s11033-023-08591-2)
Supplement: Supplementary file 1 — Supplementary Material 1 [file 11033_2023_8591_MOESM1_ESM.docx]

**Supplementary Information**

**Analysis of retest reliability for pregnant women undergoing cfDNA testing with a no-call result**

Shuqiong He^#^, Qian Zhang^#^, Meihuan Chen, Xuemei Chen, Bin Liang, Na Lin^*^, Hailong Huang^*^, Liangpu Xu^*^

Medical Genetic Diagnosis and Therapy Center, Fujian Maternity and Child Health Hospital College of Clinical Medicine for Obstetrics & Gynecology and Pediatrics, Fujian Medical University, Fujian Key Laboratory for Prenatal Diagnosis and Birth Defect, Fuzhou, China

^*^ Correspondence: Na Lin, E-mail: [linna1088@fjmu.edu.cn](mailto:linna1088@fjmu.edu.cn); Hailong Huang, E-mail: [huanghailong@fjmu.edu.cn](mailto:huanghailong@fjmu.edu.cn); Liangpu Xu, E-mail: [xiliangpu@fjmu.edu.cn](mailto:xiliangpu@fjmu.edu.cn); Tel.: 086-0591-87554929

^#^ Shuqiong He and Qian Zhang contributed equally.


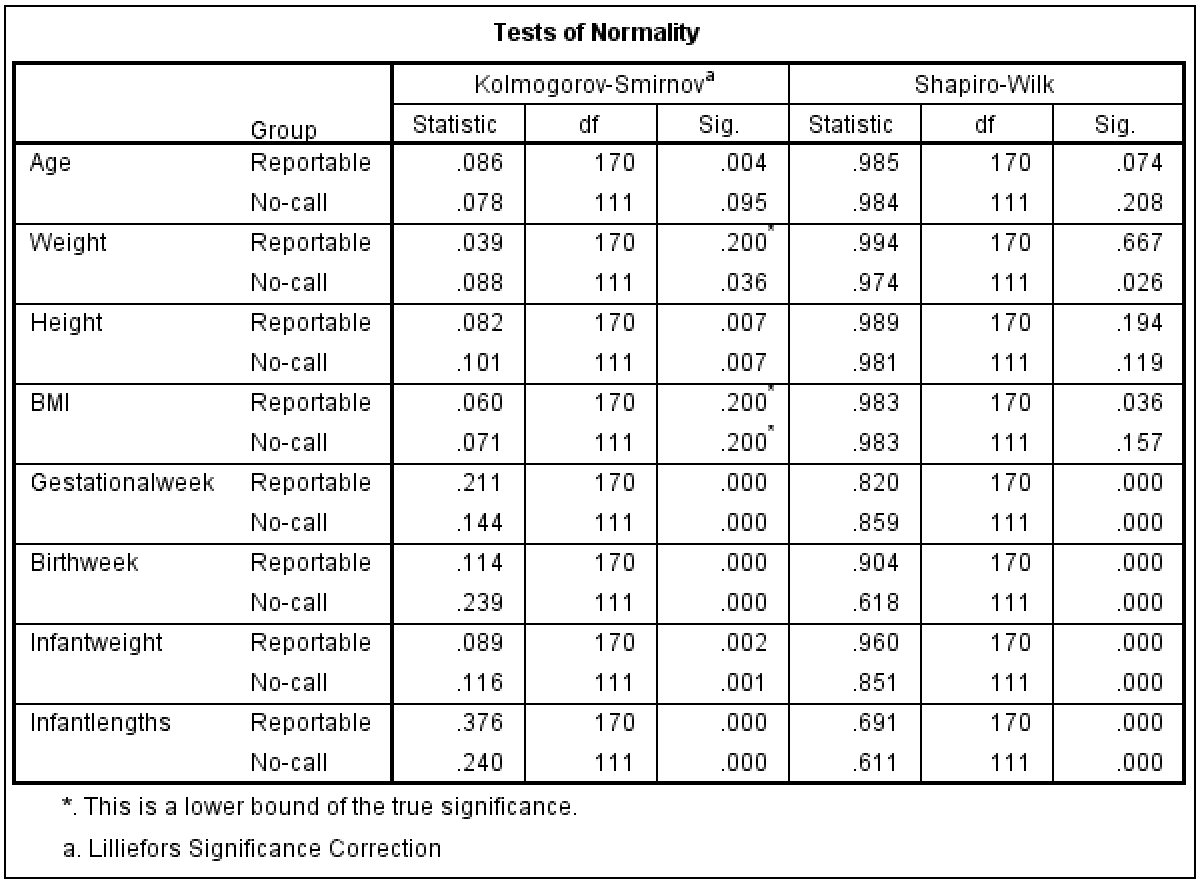


**Fig. S1** Results of the normality test


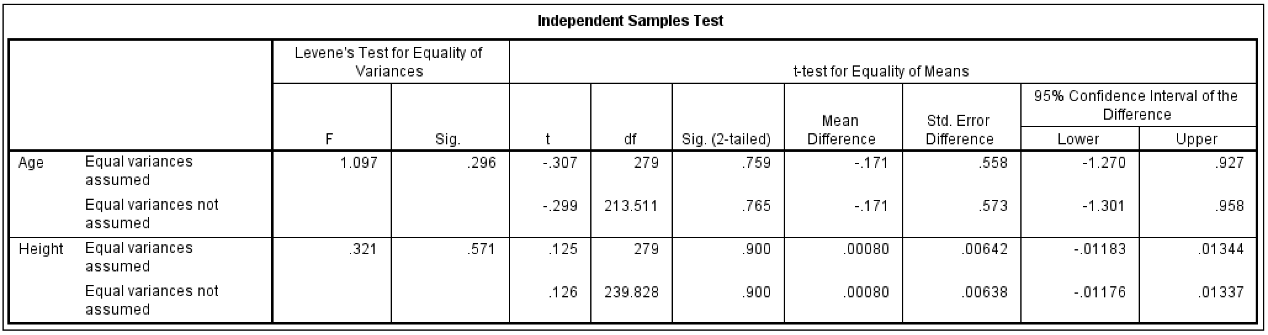


**Fig. S2** Results of the Student’s t test


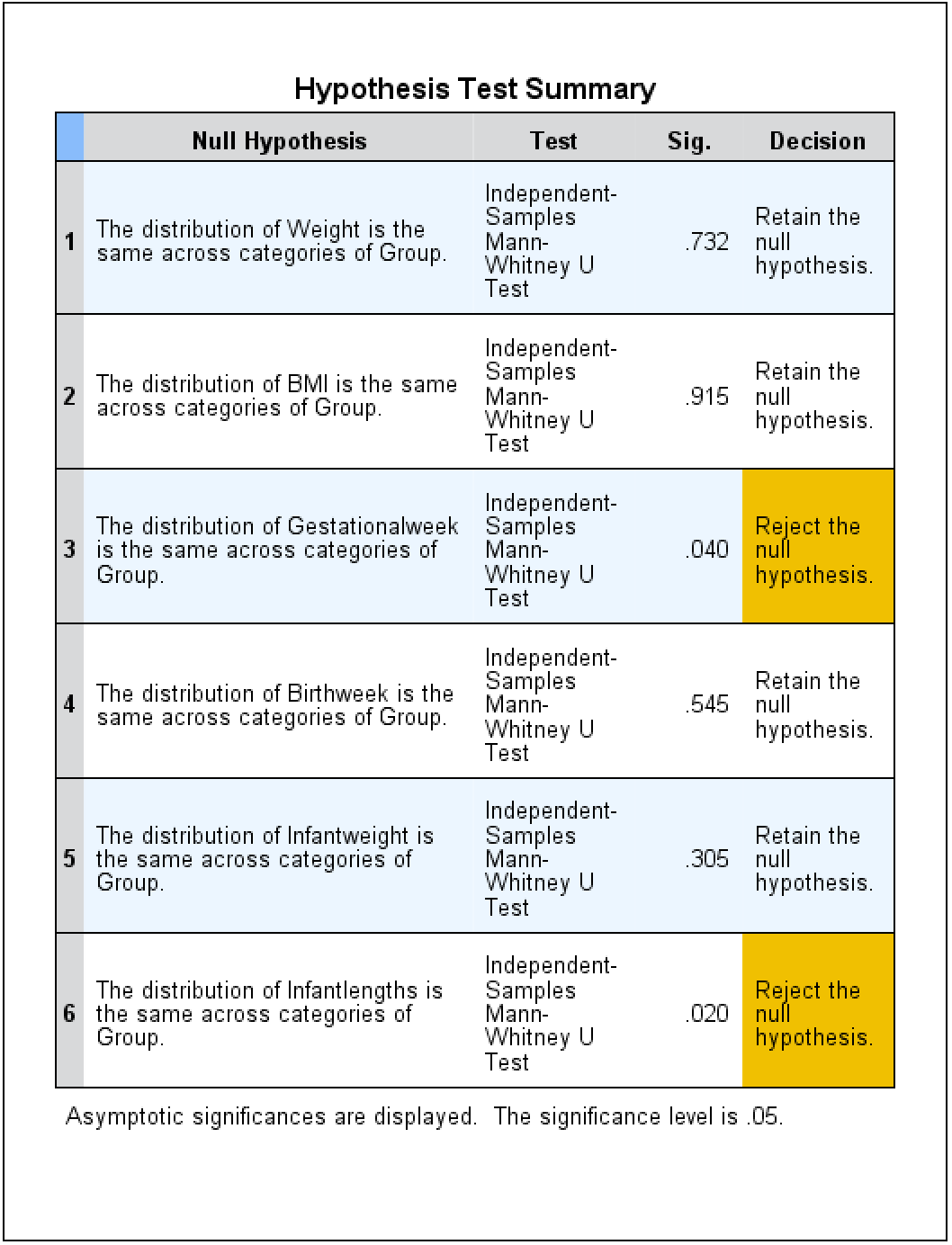


**Fig. S3** Results of the Mann-Whitney U test
